# Utility of robotic surgery for Siewert type II/III adenocarcinoma of esophagogastric junction: transhiatal robotic versus laparoscopic approach

**DOI:** 10.1186/s12893-023-02045-z

**Published:** 2023-05-16

**Authors:** Masaaki Nishi, Yuma Wada, Kozo Yoshikawa, Chie Takasu, Takuya Tokunaga, Toshihiro Nakao, Hideya Kashihara, Toshiaki Yoshimoto, Mitsuo Shimada

**Affiliations:** Department of Surgery, University of Tokushima Graduate School, 3-18-15 Kuramoto-Cho, Tokushima, 770-8503 Japan

**Keywords:** Siewert II/III, Adenocarcinoma of esophagogastric junction, Robotic surgery, Laparoscopic surgery

## Abstract

**Background:**

Robotic surgery (RS) has been rapidly adopted for gastric cancer and adenocarcinoma of the esophagogastric junction (AEG). However, the utility of RS for Siewert type II/III AEG remains unclear.

**Methods:**

Forty-one patients who underwent either transhiatal RS (*n* = 15) or laparoscopic surgery (LS) (*n* = 26) for Siewert type II/III AEG were enrolled in this study. The surgical outcomes of the two groups were compared.

**Results:**

In the entire cohort, there were no significant intergroup differences in the operative time, blood loss volume, or number of retrieved lymph nodes. The length of the postoperative hospital stay was shorter in the RS group than in the LS group (14.20 ± 7.10 days vs. 18.73 ± 17.82 days, respectively; *p* = 0.0388). The morbidity rate (Clavien–Dindo grade ≥ 2) was similar between the groups. In the Siewert II cohort, there were no significant intergroup differences in short-term outcomes. In the entire cohort, there was no significant difference between the RS and LS groups in the 3-year overall survival rate (91.67% vs. 91.48%, N.S.) or 3-year disease-free survival rate (91.67% vs. 91.78%, N.S.), respectively. Likewise, in the Siewert type II cohort, there was no significant difference between the RS and LS groups in the 3-year overall survival rate (80.00% vs. 93.33%, N.S.) or 3-year disease-free survival rate (80.00% vs. 94.12%, N.S.), respectively.

**Conclusions:**

Transhiatal RS for Siewert II/III AEG was safe and contributed to similar short-term and long-term outcomes compared with LS.

## Synopsis

This single-center study was performed to compare transhiatal robotic surgery versus laparoscopic surgery for Siewert II/III adenocarcinoma of the esophagogastric junction. Robotic surgery was safe and provided feasible short-term and long-term survival outcomes compared with laparoscopic surgery.

## Introduction

The incidence of adenocarcinoma of the esophagogastric junction (AEG) has been increasing in East Asia, as in Western countries [1, 2]. This increase in AEG is related to obesity, reflux esophagitis, smoking, and a decreased incidence of *Helicobacter pylori*infection. Surgery is the only curative treatments for AEG [3, 4].

Robotic surgery (RS) for gastrointestinal malignancy has markedly improved and provides potential benefits over conventional open or laparoscopic surgery (LS) [5–8]. RS has several surgical advantages over LS, including a three-dimensional surgical field of view, a comfortable ergonomic surgical environment, easier instrument movement, less fatigue, and less tremor filtering for operators [9, 10]. RS for gastric cancer was first reported in 2003 [11]. Subsequent clinical trials have shown that RS is a safe and reliable surgical procedure that leads to favorable short- and long-term outcomes for gastric cancer [6–13].

Based on the results of a high-quality randomized controlled trial, the transhiatal approach, which consisted of total gastrectomy with lower esophagectomy, has become a standard procedure for AEG invading the esophagus by ≤ 3 cm in Japan [14]. However, the utility of minimally invasive surgery (MIS) for Siewert II/III AEG remains controversial because of the lack of scientific evidence. Although several recent studies have suggested the utility of LS for Siewert II/III AEG [15–21], little is known about the utility of RS for Siewert II/III AEG. The clinical benefits of RS for Siewert II/III AEG remain unclear.

The present study was performed to compare transhiatal RS versus LS for Siewert II/III AEG in patients treated in a single center.

## Methods

### Patients

This single-institution retrospective cohort study included 41 eligible patients who underwent curative transhiatal MIS for primary fStage I–III, Siewert type II/III AEG at Tokushima University Hospital from May 2008 to June 2022. We excluded patients who had AEG with > 3 cm of esophageal invasion. Fifteen patients underwent transhiatal RS, and 26 patients underwent transhiatal LS. The procedure and hospitalization costs were covered by insurance for all patients. All patients provided written informed consent to undergo the surgery after receiving a detailed explanation of each surgical procedure and the associated risks. LS was conducted from 2008 to 2022 (median: 2014.5) and RS from 2018 to 2022 (median: 2019.5). The short- and long-term surgical outcomes were compared between the RS and LS groups. This study was approved in advance by the Institutional Review Board of the University of Tokushima Graduate School of Medical Science (TOCMS: 3215–1).

### Definitions of comorbidities

Definition of co-morbidity was previously reported [13]. The comorbidities evaluated in this study were defined as follows. Stroke was defined as symptomatic stroke with subsequent neurological disturbance. Renal failure was defined as treatment with dialysis or a serum creatinine concentration of ≥ 2 mg/dL. Liver cirrhosis was defined as a preoperative indocyanine green retention rate at 15 min of ≥ 15%. Cardiac disease was defined as a history of myocardial infarction, percutaneous coronary intervention, coronary artery bypass grafting, or heart failure. Pulmonary disease was defined as a percent vital capacity or forced expiratory volume in 1 s of ≤ 50%. Diabetes mellitus was defined as current insulin use.

### Surgical procedure

The stage and extent of lymphadenectomy were classified in accordance with the Japanese Classification of Gastric Carcinoma. The surgical indications and lymph node (LN) dissection were defined using the Japanese Gastric Cancer Treatment Guidelines 2021 (6th edition). The surgical techniques of RS and LS in our institute were reported previously [22–24]. We performed extended total gastrectomy (TG) with lower mediastinal LN dissection or extended proximal gastrectomy (PG) with lower mediastinal LN dissection for Siewert II/III AEG. Intraoperative upper endoscopy was routinely employed. We confirmed a negative oral margin by intraoperative fresh frozen section in all cases. The reconstruction method for TG was Roux-en-Y [25], and that for PG was esophagogastrostomy with the circular method, the Kamikawa flap method [26, 27], or modified side overlap with fundoplication by Yamashita (mSOFY) [28].

The method of statistical analysis was previously reported [13]. Values are shown as mean ± standard deviation. All statistical analyses were performed using statistical software (JMP 8.0.1.; SAS Institute, Cary, NC, USA). Clinical variables were analyzed with the chi-squared test and Wilcoxon test. Survival curves were plotted using the Kaplan–Meier method. Statistical significance was defined as *p* < 0.05.

## Results

### Patient characteristics

We enrolled 41 patients with Siewert type II/III AEG. Among these 41 patients, 26 underwent transhiatal LS and 15 underwent transhiatal RS. The detailed clinicopathological characteristics of the entire cohort are provided in Table 1. There were no significant differences in age, sex, body mass index, pT factor, pN factor, pStage, lymphatic invasion, venous invasion, tumor markers, surgical procedure, or reconstruction methods between the RS and LS groups. The technical aspects of the surgery for Siewert type II AEG are more difficult than those for gastric cancer and Siewert type III AEG with regard to lower mediastinal dissection and higher anastomosis [15, 16]. Therefore, we evaluated the surgical outcomes in Siewert II cohort. The characteristics of the patients in the Siewert II cohort are shown in Table 2; no significant intergroup differences were found.

**Table 1:** Patients’ characteristics in entire cohort

Variables	RS group (*n* = 15)	LS group (*n* = 26)	*p* value
Patient-related factors			
Age (years)	72.20 ± 11.52	66.65 ± 11.52	0.2225
Sex (male/female)	12/3	21/5	0.9523
BMI (kg/m^2^)	24.25 ± 4.09	23.42 ± 3.16	0.3299
Comorbidities			
Stroke (no/yes)	12/3	24/2	0.2460
Renal failure (no/yes)	15/0	26/0	N.S
Liver cirrhosis (no/yes)	15/0	25/1	0.4419
Cardiac disease (no/yes)	15/0	24/2	0.2703
Pulmonary disease (no/yes)	15/0	26/0	N.S
Diabetes mellitus (no/yes)	15/0	25/1	0.4419
Tumor-related factors			
pT (1/2/3/4)	5/3/5/2	17/3/5/1	0.2375
pN (0/1/2/3)	13/1/1	20/3/3	0.7501
fStage (I/II/III)	8/6/1	18/6/2	0.5159
Lymphatic invasion (− / +)	9/6	16/10	0.9225
Venous invasion (− / +)	7/8	14/12	0.6578
CEA (< 5/ ≥ 5 ng/mL)	14/1	25/1	0.6863
CA19-9 (< 37/ ≥ 37 IU/mL)	14/1	25/1	0.6863
Treatment-related factors			
Procedure (TG/PG)	13/2	18/8	0.2105
Reconstruction			0.2453
Roux-en-Y esophagojejunostomy	13	18	
mSOFY	2	2	
Kamikawa flap	0	2	
Esophagogastrostomy (circular)	0	4	
Neoadjuvant chemotherapy (no/yes)	1/14	0/26	0.1826
Adjuvant chemotherapy (no/yes)	12/3	23/3	0.4603

Data are shown as mean ± standard deviation or n

*LS* Laparoscopic surgery, *RS* Robotic surgery, *BMI* Body mass index, *CEA* Carcinoembryonic antigen, *CA19-9* Carbohydrate antigen 19–9, *TG* Total gastrectomy, *PG* Proximal gastrectomy, *mSOFY* Modified side overlap with fundoplication by Yamashita, *N.S.* Not significant

**Table 2:** Patients’ characteristics in Siewert II AEG cohort

Variables	RS group (*n* = 8)	LS group (*n* = 17)	*p* value
Patient-related factors			
Age (years)	75.38 ± 13.22	64.25 ± 12.31	0.0504
Sex (male/female)	6/2	13/4	0.9360
BMI (kg/m^2^)	23.63 ± 4.37	23.12 ± 3.07	0.6412
Comorbidities			
Stroke (no/yes)	6/2	15/2	0.3998
Renal failure (no/yes)	8/0	17/0	N.S
Liver cirrhosis (no/yes)	8/0	16/1	0.4419
Cardiac disease (no/yes)	8/0	15/2	0.2703
Pulmonary disease (no/yes)	8/0	17/0	N.S
Diabetes mellitus (no/yes)	8/0	17/0	N.S
Tumor-related factors			
pT (1/2/3/4)	3/0/4/1	9/2/5/1	0.5419
pN (0/1/2/3)	7/0/1	12/3/2	0.4451
fStage (I/II/III)	3/4/1	10/5/2	0.5716
Lymphatic invasion (− / +)	4/4	9/8	0.8908
Venous invasion (− / +)	3/5	6/11	0.9146
CEA (< 5/ ≥ 5 ng/mL)	7/1	17/0	0.6863
CA19-9 (< 37/ ≥ 37 IU/mL)	7/1	16/1	0.5694
Treatment-related factors			
Procedure (TG/PG)	6/2	11/6	0.2105
Reconstruction			0.4641
Roux-en-Y esophagojejunostomy	6	11	
mSOFY	2	2	
Kamikawa flap	0	1	
Esophagogastrostomy (circular)	0	3	
Neoadjuvant chemotherapy (no/yes)	7/1	17/0	0.1368
Adjuvant chemotherapy (no/yes)	5/3	15/2	0.1335

Data are shown as mean ± standard deviation or n

*AEG* Adenocarcinoma of the esophagogastric junction, *LS* Laparoscopic surgery, *RS* Robotic surgery, *BMI* Body mass index, *CEA* Carcinoembryonic antigen, *CA19-9* Carbohydrate antigen 19–9, *TG* Total gastrectomy, *PG* Proximal gastrectomy, *mSOFY* Modified side overlap with fundoplication by Yamashita, *N.S.* Not significant

### Short‑term outcomes

Tables 3 and 4 summarize the operative time, blood loss volume, drain amylase content (d-AMY) on postoperative day 1, number of retrieved LNs, length of postoperative hospital stay, and morbidity rate (Clavien–Dindo grade ≥ 2) in the entire cohort and the Siewert II cohort, respectively. In the entire cohort, there were no significant intergroup differences in the operative time (RS: 382.20 ± 77.23 min vs. LS: 351.34 ± 61.01 min, p = 0.2612), blood loss volume (RS: 75.67 ± 78.40 mL vs. LS: 71.69 ± 104.28 mL, *p* = 0.3079), d-AMY on postoperative day 1 (RS: 361.06 ± 547.24 IU/L vs. LS: 453.38 ± 484.13 IU/L, *p* = 0.3939), or number of retrieved LNs (RS: 30.80 ± 14.67 vs. LS: 27.38 ± 14.57, *p* = 0.3638). The length of the postoperative hospital stay was shorter in the RS group than in the LS group (RS: 14.20 ± 7.10 days vs. LS: 18.73 ± 17.82 days, *p* = 0.0388). The morbidity rate (Clavien-Dindo grade ≥ 2.) was similar (LS15.38% vs RS 6.67%, *p* = 0.4113). No patients in the RS group developed intra-abdominal infectious complications, including anastomotic leakage. In the Siewert II cohort, there were no significant intergroup differences in the operative time (RS: 402.62 ± 103.75 min vs. LS: 349.59 ± 58.79 min, *p* = 0.1801), blood loss volume (RS: 64.00 ± 61.91 mL vs. LS: 77.71 ± 122.95 mL, *p* = 0.4285), d-AMY on postoperative day 1 (RS: 238.00 ± 198.43 IU/L vs. LS: 275.52 ± 235.68 IU/L, *p* = 0.7049), number of retrieved LNs (RS: 26.50 ± 9.66 vs. LS: 24.82 ± 11.15, *p* = 0.6827), or length of the postoperative hospital stay (RS: 15.13 ± 7.73 days vs. LS: 20.11 ± 21.70 days, *p* = 0.4091). The morbidity rate (Clavien–Dindo grade ≥ 2) was comparable between the groups (RS: 12.50% vs. LS: 17.64%, *p* = 0.7433).

**Table 3:** Short-term surgical outcomes in entire cohort

Variables	RS group (*n* = 15)	LS group (*n* = 26)	*p* value
Operative time (min)	382.20 ± 77.23	351.34 ± 61.01	0.2612
Docking time (min)	16.60 ± 4.00	N.A	N.A
Console time^a^ (min)	206.20 ± 49.95	N.A	N.A
Operative blood loss (mL)	75.67 ± 78.40	71.69 ± 104.28	0.3079
d-AMY on POD 1 (IU/L)	361.06 ± 547.24	453.38 ± 484.13	0.3939
Retrieved LNs (n)	30.80 ± 14.67	27.38 ± 14.57	0.3638
Postoperative hospital stay (days)	14.20 ± 7.10	18.73 ± 17.82	0.0388
Morbidity^b^ (no/yes)	14/1	22/4	0.4113
Grade II	0	3	
Grade III	1	1	
Grade IV	0	0	
Grade V	0	0	
Complications^b^			
Pancreatic fistula	0	1	
Ileus	1	0	
Anastomotic leakage	0	2	
Intraluminal bleeding	0	1	

Data are shown as mean ± standard deviation or n

*LS* Laparoscopic surgery, *RS* Robotic surgery, *d-AMY* Drain amylase content, *POD* Postoperative day, *LN* Lymph node, *N.A.* Not available

^a^From docking to gastrectomy

^b^Clavien–Dindo grade ≥ 2

**Table 4:** Short-term surgical outcomes in Siewert II AEG cohort

Variables	RS group (*n* = 8)	LS group (*n* = 17)	*p* value
Operative time (min)	402.62 ± 103.75	349.59 ± 58.79	0.1801
Docking time (min)	15.50 ± 4.28	N.A	N.A
Console time^a^ (min)	200.88 ± 67.11	N.A	N.A
Operative blood loss (mL)	64.00 ± 61.91	77.71 ± 122.95	0.4285
d-AMY on POD 1 (IU/L)	238.00 ± 198.43	275.52 ± 235.68	0.7049
Retrieved LNs (n)	26.50 ± 9.66	24.82 ± 11.15	0.6827
Postoperative hospital stay (days)	15.13 ± 7.73	20.11 ± 21.70	0.4091
Morbidity^b^ (no/yes)	7/1	14/3	0.7433
Grade II	0	2	
Grade III	1	1	
Grade IV	0	0	
Grade V	0	0	
Complications^b^			
Ileus	1	0	
Anastomotic leakage	0	2	
Intraluminal bleeding	0	1	

Data are shown as mean ± standard deviation or n

*AEG* Adenocarcinoma of the esophagogastric junction, *LS* Laparoscopic surgery, *RS* Robotic surgery, *d-AMY* Drain amylase content, *POD* Postoperative day, *LN* Lymph node, *N.A.* Not available

^a^From docking to gastrectomy

^b^Clavien–Dindo grade ≥ 2

### Long‑term outcomes

**Fig. 1:**
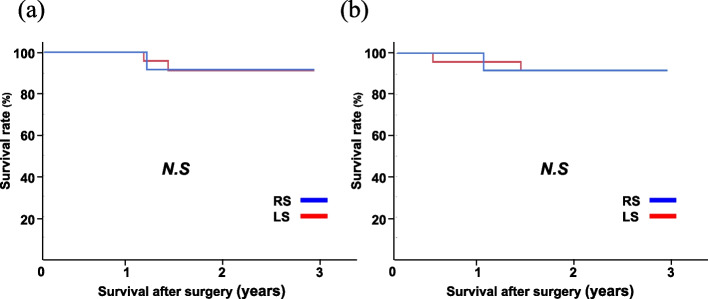
Long-term outcomes in the entire cohort. **a** Overall survival. **b** Disease-free survival. RS, robotic surgery; LS, laparoscopic surgery; N.S., not significant

**Fig. 2:**
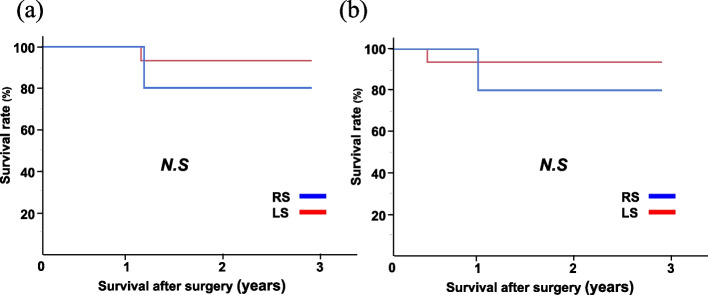
Long-term outcomes in the Siewert type II cohort. **a** Overall survival. **b** Disease-free survival. RS, robotic surgery; LS, laparoscopic surgery; N.S., not significant

The follow-up endpoint was July 2022. The median follow-up period was 40.33 months. In the entire cohort, there was no significant difference between the RS and LS groups in the 3-year overall survival rate (91.67% vs. 91.48%, N.S.) or 3-year disease-free survival rate (91.67% vs. 91.78%, N.S.), respectively (Fig. 1a, b). Likewise, in the Siewert type II cohort, there was no significant difference between the RS and LS groups in the 3-year overall survival rate (80.00% vs. 93.33%, N.S.) or 3-year disease-free survival rate (80.00% vs. 94.12%, N.S.) (Fig. 2a, b).

## Discussion

Robotic systems have been widely applied for several types of malignancy. The current study demonstrated the safety and efficacy of transhiatal RS compared with transhiatal LS for Siewert II/III AEG in patients from a single center.

The currently available surgical procedures for Siewert II/III AEG are generally divided into three types: Ivor Lewis esophagectomy, extended PG, and extended TG [29]. The surgical approaches for AEG vary widely and include open abdominal, thoracic, laparoscopic, thoracoscopic, and robotic surgeries [14–21, 29–36]. The Japan Clinical Oncology Group 9502 study suggested that the transhiatal approach had a survival benefit over the left thoracoabdominal approach for AEG with < 3 cm of esophageal invasion [14]. Mine et al. investigated the appropriate proximal margin and found that proximal margin lengths of > 2 cm in resected specimens seemed to promote better survival for patients with Siewert II/III AEG [32]. A nationwide multicenter study of AEG in Japan revealed the lymphatic metastatic rate [33]. The study showed that dissection of LN stations 1, 2, 3, 7, 8a, and 119 is recommended for AEG with esophageal involvement of ≤ 2.0 cm using a transhiatal approach [33]. Furthermore, additional dissection of LN station 110 should be performed for AEG with esophageal involvement of 2.1 to 4.0 cm [33]. Finally, based on these well designed studies, a transhiatal approach is adopted for AEG with ≤ 4 cm invasion [33].

The Japanese guideline recently provided a weak recommendation for PG, not TG, for the treatment of AEG. AEG with a tumor diameter of ≤ 4 cm does not require dissection of LN stations 4d, 5, and 6 with regard to the metastatic rate and dissection index [2]. In our institution, PG is indicated for distal invasion not exceeding 4 cm. In contrast, the above-mentioned study showed that the LN metastatic rate for AEG with > 5 cm of gastric invasion from the esophagogastric junction was elevated to 20% at LN stations 4sb, 4d, 5, and 6 [33]. In cases involving > 5 cm of gastric invasion from the esophagogastric junction, TG is recommended to obtain a sufficient distal margin and adequate LN dissection.

When performing reconstruction, esophagojejunal or esophagogastric anastomosis is technically difficult. Several reconstruction procedures have been proposed for esophagojejunostomy or esophagogastrostomy, including the circular method, the overlap method, functional end-to-end anastomosis, the Kamikawa flap, the tri double-flap hybrid method, SOFY, and side-overlap esophagogastric tube reconstruction [25–28, 34, 35]. We have used the circular method with a transoral anvil delivery system for esophagojejunostomy in robotic or laparoscopic TG. The dissection length to the proximal esophagus can be minimized when using the circular method. Especially in AEG, the linear method requires 4 to 5 cm of dissection to the proximal esophagus in addition to the 2-cm proximal margin. Long proximal dissection with higher anastomosis is difficult and may lead to critical anastomotic complications. Fortunately, we have experienced no anastomotic leakage when performing esophagojejunostomy in patients with AEG.

MIS for AEG is challenging because of the difficulty of sufficient LN dissection and safe anastomosis. Several Eastern studies have suggested both feasible short-term surgical outcomes and long-term oncological safety of LS compared with surgery for Siewert II/III AEG [15–21]. Sugita et al. reported that LS resulted in less intraoperative blood loss and better overall survival [15, 16]. A meta-analysis showed that LS contributed to less intraoperative blood loss and a shorter postoperative hospital stay, and the overall postoperative complications of LS were significantly lower than those of open surgery [21]. Based on these results, LS is becoming a promising option for Siewert II/III AEG.

For gastric cancer, several clinical trials have shown that RS is a reliable surgical procedure that leads to favorable short- and long-term outcomes [6–8, 12, 13]. Compared with LS, RS reduces the intraoperative blood loss volume, morbidity, and learning curve; increases the number of retrieved LNs; and provides similar long-term outcomes. We previously reported the short-term superiority of RS over LS for gastric cancer[13]. One prospective randomized controlled trial compared the short-term efficacy of robotic versus laparoscopic distal gastrectomy and showed that RS achieved a lower morbidity rate, faster recovery, a milder inflammatory response, and improved lymphadenectomy [6]. However, the utility of RS for Siewert II/III AEG remains controversial because of the lack of scientific evidence. Some experienced surgeons have demonstrated their robotic surgical technique [31, 36]. Ikoma et al. introduced the PG with fluorescent sentinel lymphatic mapping performed by injecting indocyanine green solution for AEG [31].

Our results confirmed these feasible short-term and long-term outcomes of RS compared with LS for Siewert II/III AEG. The original utility of the robotic system enabled us to perform a safe operation. We believe that RS has utility for both Siewert II/III AEG and gastric cancer and has some surgical advantages including high-definition three-dimensional vision, stable vision, flexible instruments, fluorescent-image guidance with use of indocyanine green, careful LN dissection, and easier intracorporeal hand-sewing in the lower mediastinum. However, the technological advantages of RS did not result in significant superiority of surgical outcomes in the current study. RS is one subtype of MIS and is based on LS. The surgical procedures of LS and RS for AEG were standardized in the early period, and this early standardization enabled us to perform a safe operation.

The present study had some limitations. First, this was a nonrandomized, single-center, retrospective study of a small number of patients. Second, the learning curve might have affected the surgical outcomes. Finally, patient-related outcomes such as quality of life after surgery were not included in the surgical outcomes. Multicenter randomized controlled trials with larger sample sizes are warranted to elucidate the real benefit of RS for Siewert II/III AEG.

In conclusion, the present study confirmed that RS is a safe procedure and provides feasible short-term and long-term outcomes compared with LS for Siewert II/III AEG.

## Data Availability

Raw data were generated at Tokushima University. Derived data supporting the findings of this study are available from the corresponding author on request.
